# Policy and practice implications of contextual understanding of - and tools to address - mental health and psychosocial support needs in Sierra Leone

**DOI:** 10.3389/fpsyt.2025.1419448

**Published:** 2025-02-05

**Authors:** Alastair Ager, Rebecca Horn, Abdulai Bah, Haja Wurie, Mohamed Samai

**Affiliations:** ^1^ NIHR Research Unit on Health in Situations of Fragilty, Institute for Global Health and Development, Queen Margaret University, Edinburgh, United Kingdom; ^2^ NIHR Research Unit on Health in Situations of Fragility, College of Medicine and Allied Health Sciences, University of Sierra Leone, Freetown, Sierra Leone; ^3^ Ministry of Health and Sanitation, Freetown, Sierra Leone

**Keywords:** mental health, policy, culture, psychometric measurement, psychological assessment, impact

## Abstract

The last two decades have seen increased awareness of the impact of mental health issues on the population of Sierra Leone. Local capacity to respond to these needs is severely limited. In 2017, the Ministry of Health and Sanitation (MoHS) worked with staff of the College of Medicine and Allied Health Sciences (COMAHS – part of the University of Sierra Leone) and Queen Margaret University (QMU) in Edinburgh – and other stakeholders, including members of the Mental Health Coalition Sierra Leone – to define a research agenda that would support the development of community-based mental health and systems support in the community. This paper summarizes work over the course of the following six years in relation to this agenda, and indicates its relevance to ongoing and planned service developments. In terms of research advance, studies have – through participatory and ethnographically-informed methods – identified both local idioms and social determinants of distress and mapped health seeking pathways and barriers to care. This information was utilized in the development and validation of two culturally appropriate measures: the Sierra Leone Psychological Distress Scale (to assess mental health and psychosocial needs at the community level) and the Sierra Leone Perinatal Psychological Distress Scale (to identify common perinatal mental disorder in amongst pregnant and lactating mothers). For this latter population, a culturally adapted form of a problem solving intervention delivered through existing mother-to-mother supports has been shown to be feasible, acceptable and potentially effective. This work has major policy and practice implications, and early evidence of uptake is noted. This includes mental health capacity development through the online availability of training guides for the developed assessment scales and plans for incorporation of material regarding idioms and social determinants of distress in pre-and post-professional training curriculum. In terms of community-based initiatives, there has been evidence of uptake from the Mental Health Coalition Sierra Leone. In terms of policy, findings reinforce key principles regarding community-based provision, integration of mental health care into primary health care, and actions to reduce stigma associated with mental health.

## Introduction

The last two decades have seen increased awareness of the impact of mental health issues on the population of Sierra Leone. Despite the general lack of population health surveys (1), the mental health impacts of the 1991-2002 civil war and the Ebola outbreak of 2014-16 (2, 3) and, lately, the escalating drug use amongst youth (1) have been noted as considerable.

Local capacity to respond to these needs is severely limited. There have been important policy and practice developments, notably the development of the National Mental Health Strategy in 2010, the founding of the Mental Health Coalition in 2011, and the formal launch of the national Mental Health Policy and Plan in 2012 (4). These, together with the experience of Ebola and, subsequently, the COVID-19 pandemic, have focused attention on staff training to strengthen the provision of psychosocial support, notably through the scaling up of PFA and mhGAP trainings. Initiatives such as the King’s Sierra Leone Partnership (5) have supported a range of relevant service developments in relation to district mental health and psychosocial units, initial training of mental health nurses, and continuing professional development in psychiatry for medical officers, mental health nurses and other cadres.

These developments have served to address the treatment gap for mental health, which had been estimated in 2005 as above 90% (4). However, such is the magnitude of that gap and the challenges in mobilizing and retaining skilled human resources to address it (1), that there is increased recognition that a substantial element of mental health response needs to be based on community resources (6). Utilizing community based resources as the foundation for mental health service provision is the general direction of travel in global mental health policy (reflected, for example, in the approach of the World Health Organization’s Special Initiative for Mental Health). Common signs of distress such as anxiety or low mood are associated with more total disability at a population level than diagnostically defined mental disorders, and early identification of these issues leads to better outcomes. This is especially important in Sierra Leone where there are limited formal mental health services, and there is a reluctance to access such services until the situation has become very severe.

The Mental Health Coalition Sierra Leone (MHCSL) has played a significant role in promoting and strengthening community-based approaches to psychosocial support and mental health provision. The MHCSL is a network of organizations and individuals working together to improve the mental health and well-being of people in Sierra Leone. The coalition has consistently advocated for the inclusion of mental health issues in Sierra Leone’s national policies and programmes, including the Mental Health Policy and Strategic Plan, launched in 2012, and the updated version in 2019 (7). The coalition advocated for the development of mental health clinics and the provision of psychosocial support to survivors of the Ebola virus disease outbreak, and continues to advocate for the training and supporting of mental health professionals and other stakeholders. The MHCSL acts as an advisory and monitoring body on mental health issues in Sierra Leone and has contributed to the establishment of a national mental health steering committee within the Ministry of Health and Sanitation. The coalition holds annual conferences that bring together stakeholders from within the country and international participants to discuss mental health and related topics (1, 8).

In 2017, the Ministry of Health and Sanitation (MOHS) worked with staff of the College of Medicine and Allied Health Sciences (COMAHS – part of the University of Sierra Leone) and Queen Margaret University (QMU) in Edinburgh – and other stakeholders, including members of the MHCSL – to define a research agenda that would support the development of community-based mental health and systems support in the community. Informed by participatory community level meetings and consultations with health providers in Freetown and Bombali district, this agenda identified the need for (i) understanding cultural idioms of distress and explanatory models of mental illness; (ii) investigating the prevalence of mental health problems using culturally-appropriate measures; (iii) assessing the impact of mhGAP training on knowledge, attitude and practices of health professionals towards mental, neurological and substance use disorders; and (iv) integrating mental health services into perinatal care in Sierra Leone.

## Research advance

This paper summarizes work over the course of the following six years in relation to this agenda, and indicates its relevance to ongoing and planned service developments. Discussion focuses principally on the first two and final points of the identified agenda, with plans to assess impact of mhGAP trainings not being pursued due to a range of logistical challenges.

### Mapping health seeking pathways and barriers to care

**Figure 1:**
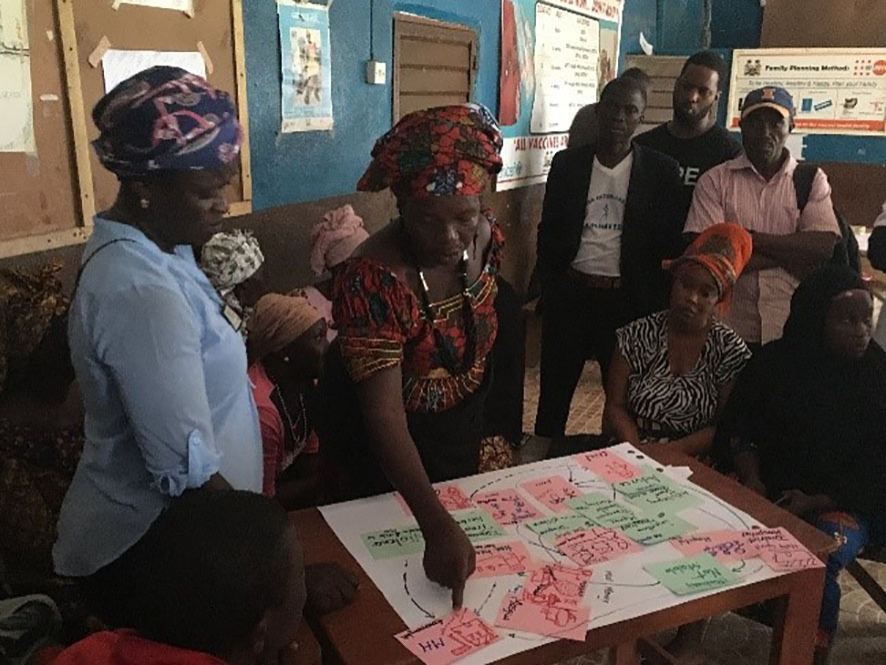
Participatory group discussion with local community members in Bombali District.

**Figure 2:**
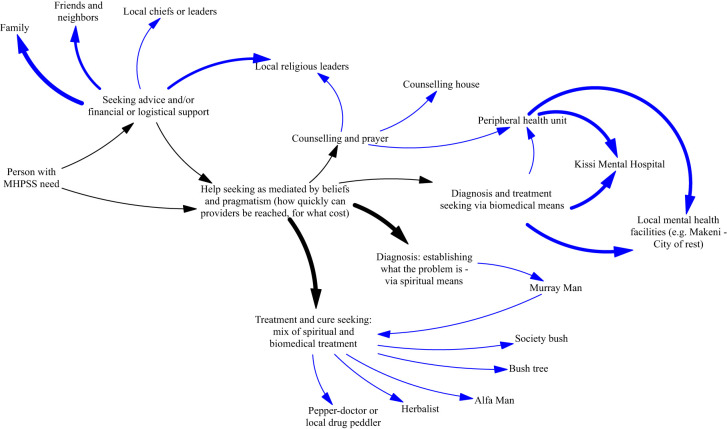
Accounts of health-seeking reported in participatory discussions (width of arrows signaling frequency of reports).

The participatory meetings that served to inform the initial research agenda provided valuable insight to health seeking pathways with regard to mental ill health and the barriers that existed to receiving care (see **Figure 1**). Informed by the methodology of Group Model Building (9), members of local communities in Freetown and Bombali with lived experience of mental health issues – and separate meetings with health providers - informed the analysis of the available ‘system for mental health’ shown in **Figure 2**. This emphasized the plurality of treatment pathways, with widespread acceptance of the potential relevance of social, traditional and allopathic resources in addressing mental health issues, but the major financial, logistical and cultural barriers that constrained access to each of them.

### Identifying idioms of distress

With the experience of psychological distress so clearly embedded within community understandings of mental health issues, a deeper understanding of the language and concepts people draw upon to describe it is clearly warranted. Work was conducted in four districts to elicit the local terms and understanding that served to communicate distress in its differing forms (10). The only consistent ‘syndrome’ identified was a general concept of *crase*, which referred to psychosis-related behaviors but also a wide range of other signs of distress. No consensus on locally defined concepts for mild-moderate forms of mental disorder was identified, with people using multiple overlapping signs and terms to indicate psychological distress. This supports viewing mental health problems in Sierra Leone on a ‘continuum of distress’ rather than as discrete categories. This approach is coherent with opportunities for prevention and response in Sierra Leone which do not focus primarily on formal healthcare service providers but rather involve a range of community-based actors. It also encourages attention to the identification of milder signs of distress with a view to early response and prevention of more severe mental health problems.

Given evidence of particular mental health needs of women in the perinatal period of motherhood, a further strand of work elicited idioms of distress particularly associated with this sub-population (11, in review). This work identified idioms such as *pwel at* (feeling sad and discouraged) and *mi maynd no get pis* (thinking too much, lack of peace of mind) as amongst the most consistent expressions of distress for pregnant and lactating mothers. Such distress was commonly related to marital/partner disharmony, gender norms, problems with in-laws, poverty, ill health and lack of basic amenities. Distress generally led to consultation with religious leaders, herbalists, friends, neighbors and family members, with common coping strategies involving prayers, sleeping, listening to music and use of alcohol to forget about problems.

### Development of the Sierra Leone Psychological Distress Scale and Sierra Leone Perinatal Psychological Distress Scale

Awareness of local idioms of distress is clearly useful for health workers and others seeking to provide mental health and psychosocial support in this context. However, systematic documentation of these also provides the opportunity to create a screening measure that can identify those in need of particular attention.

The Sierra Leone Psychological Distress Scale (SLPDS) was developed on the basis of fieldwork in four districts, and provides a robust and accessible measure for wide use in Sierra Leone (12). The 18-item distress scale consists of three subscales, all of which showed good internal consistency. These sub-scales relate respectively to high emotional arousal (fear, anxiety, anger etc.), passive signs of distress (specifically hopelessness and feelings of worthlessness and withdrawal) and physical and behavioral issues (including sleep, appetite and engaging with other people). The scale is accompanied by a gendered measure of ability to carry out daily tasks - a Function scale - as an indication of the severity of distress. The SLPDS is not intended as an individual screening tool, but as a measure which can be used at community-level to assess mental health and psychosocial needs in order to support the planning of service provision and the evaluation of initiatives designed to improve mental health and psychosocial wellbeing.

**Figure 3:**
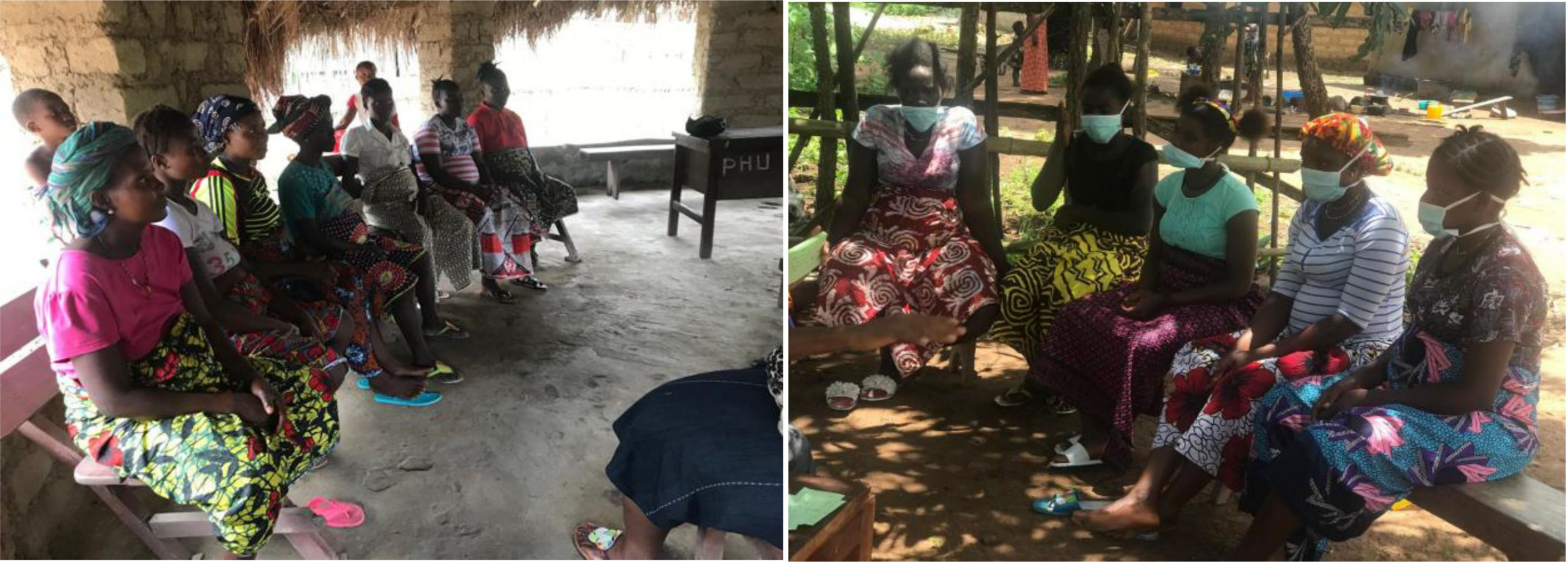
Focus Group Discussions with perinatal women during the development of the SLPPDS (left, before COVID 19, and right, during the COVID 19 pandemic).

The Sierra Leone Perinatal Psychological Distress Scale was developed through a similar process (13). The SLPPDS is a ten-item scale that effectively predicts a woman presenting with perinatal psychological distress, and thus offers strong potential as a screening tool. A five-item function scale assesses impact of distress on work and social functions. While countries with more resources use established tools like the PHQ-9 to screen for issues like depression, these may not work well in Sierra Leone due to cultural differences. The tool covers a range of symptoms including sadness, irritability, sleep disturbance, feelings of shame, and thoughts of harming a partner. The total score ranges from 0-30, with a score of 8 or higher suggesting a case of potential psychological distress. This new tool can help improve how mental health issues are identified and addressed, ultimately benefiting mothers and enhancing support services (see **Figure 3**).

### Identifying social determinants of psychological distress

The development of a nationally valid measure of psychological distress provides a basis for analysis of factors associated with higher scoring on the measure. Identification of such factors not only informs the identification of high risk groups, but wider understanding of preventive strategies. Accordingly, data from the SLPDS validation study was used to identify predictors of higher levels of psychological distress in Sierra Leone (14, 15). Analysis suggested that family conflict and inability to afford basic needs made the greatest contribution to psychological distress. Gender differences were evident: factors most predictive of men’s psychological distress were severe sickness or injury and being unable to afford basic needs; for women, the main predictive factors were family conflict, perceived poor physical health and inability to afford basic needs.

### Trialling community based interventions

**Figure 4:**
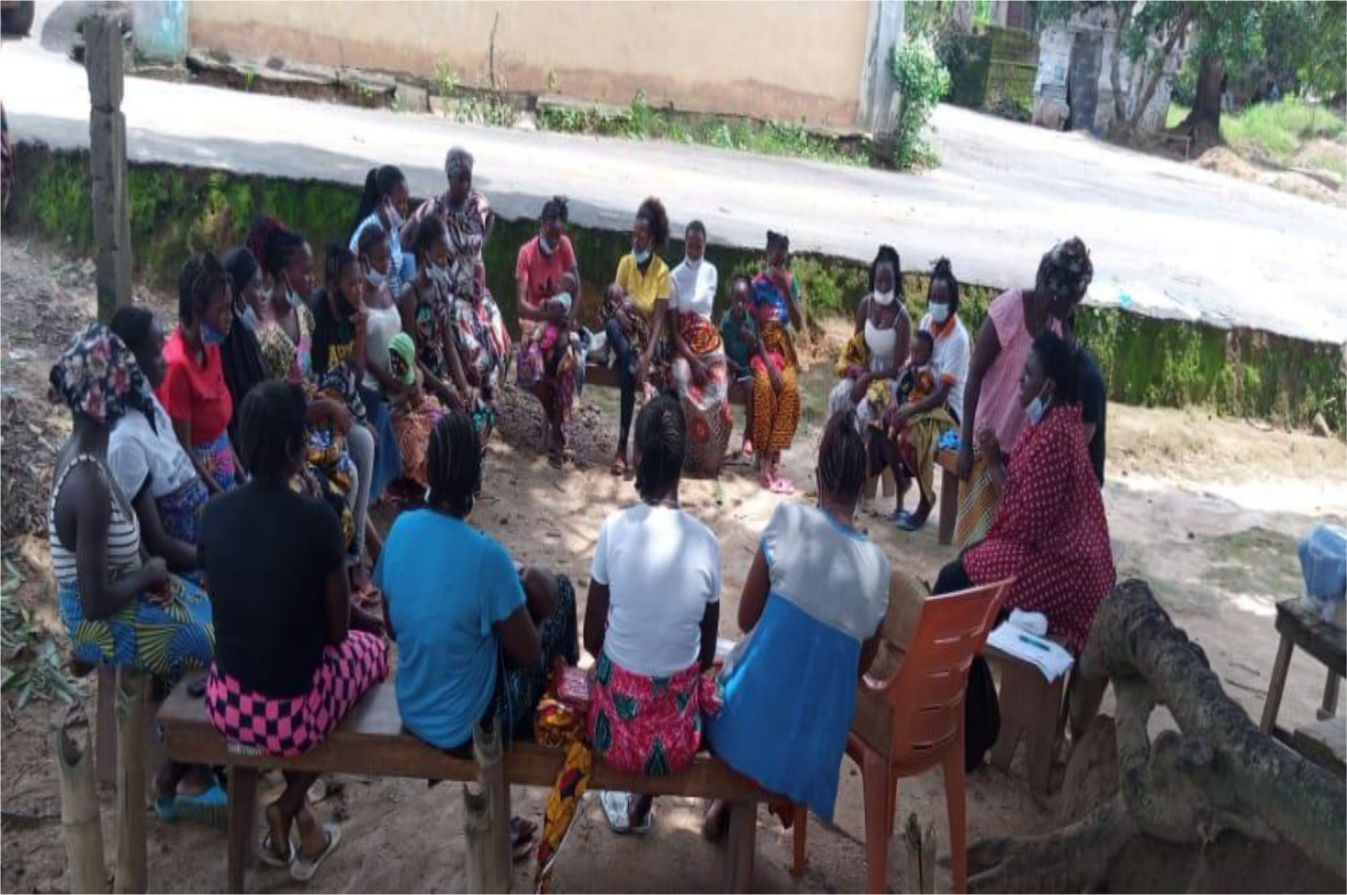
Group meeting (‘col at sacul’) at the end of the four week intervention for pregnant women and new mothers experiencing psychological distress.

In the case of perinatal women, our work has advanced to the stage of trialling an intervention to support pregnant and lactating mothers who report high levels of distress on the scale (16, in press). A culturally adapted form of the Friendship Bench problem solving intervention incorporating psychoeducation effectively benefitted perinatal women experiencing psychological distress. Delivered by lay women with no prior mental health training, the intervention demonstrated that with supportive supervision it is feasible to equip women with low literacy levels at community level to provide effective mental health support. Delivered over a four-week period, the intervention is feasible for scalable delivery in the country. Further, integrating such interventions in the routine activities of mother-to-mother support groups and addressing local idioms of distress stands to reduce stigma of addressing perinatal distress (see **Figure 4**).

## Policy and practice uptake

### Healthcare staff training and curriculum development

Given cultural practices and beliefs play such a significant role in shaping perceptions of mental health and seeking help for emotional distress in Sierra Leone it is crucial to incorporate these concepts into healthcare staff training and curriculum development to ensure that healthcare providers can provide culturally sensitive and effective care for relevant populations. In addition to addressing the cultural aspects of perinatal psychological distress, healthcare staff training and curriculum development in Sierra Leone can also usefully focus on building skills related to mental health assessment and treatment (17). Healthcare providers can be trained to use the tools to identify those at risk of psychological distress and to provide evidence-based interventions when appropriate. Training programmes can also focus on strategies to address the stigma associated with mental health and to promote mental health awareness and education within communities (18). These measures will ensure healthcare providers can improve their cultural competency and sensitivity, and provide more effective and accessible care.

**Figure 5:**
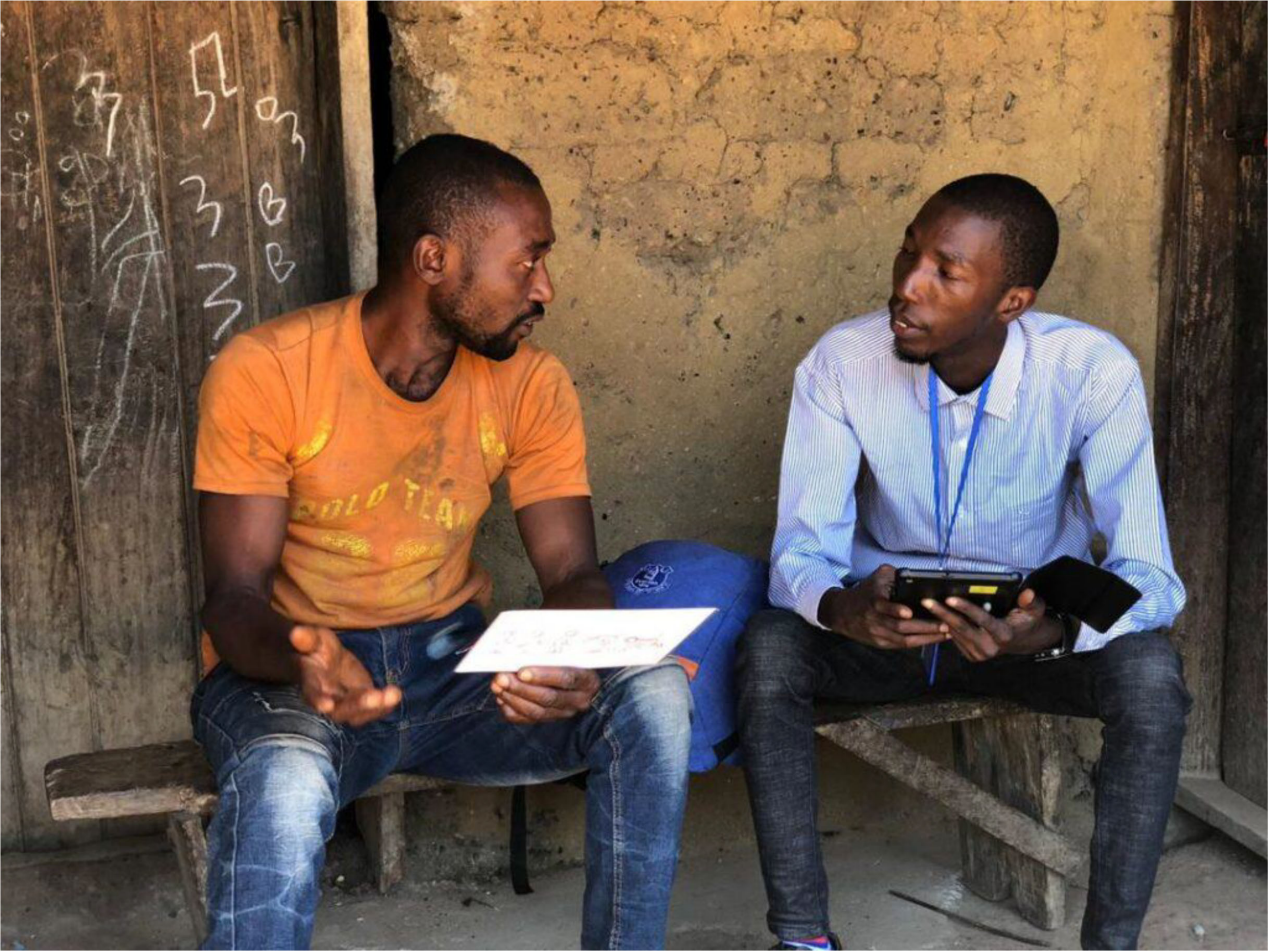
Training guides for the SLPDS feature guided role plays to clarify key practice points.

To date, the above research findings have been shared through a range of national mechanisms, including the agenda setting stakeholder engagement meeting after initial scoping work, periodic meetings with the Mental Health Coalition (including presenting at their national conference), and update meetings with the Director of NCDs and Mental Health at the MoHS. Training guides for the SLPDS are available online^1^ and have been utilized in a national stakeholder briefing event in Freetown in April 2023 (see **Figure 5**). There are plans to include material regarding idioms of distress and screening in the pre-and post-professional training curriculum at COMAHS, including for community nursing, pharmacy and clinical officers. For example, a recent curriculum review conducted by COMAHS resulted in the addition of a sexual and reproductive module for medical students during their clinical years, with a particular emphasis on psychosocial wellbeing.

### Community development initiatives

The MHCSL was an active participant in the development of the research agenda and in the national stakeholder briefing event in April 2023. MHCSL has continued working to create a better mental health system through collaboration, advocacy, and awareness-raising. The coalition has conducted community outreach programs, radio broadcasts, and training workshops for health workers, civil society organizations, and community leaders. These efforts have helped to increase awareness about mental health and reduce the stigma and discrimination that many people with mental illness face. Insights from the research will assist MHCSL’s goal to empower stakeholders by providing resources and advocacy, particularly service users, to voice their own priorities and needs. Another MHCSL goal - to increase awareness about mental health and promote mental wellness in the general population (8) – is are also clearly supported by research outputs. The MHCSL 2024 conference included a session on the SLPDS to raise awareness of the tool and promote its use. Integration of use of the SLPPDS in settings like the Rainbow Centre represents a significant step towards improving the mental health care of sexual violence survivors in Sierra Leone. By identifying and addressing psychological distress early, non-healthcare and healthcare providers can play a crucial role in the recovery and empowerment of these individuals, ultimately contributing to their overall well-being and reintegration into society. Outside of the mental health sector, work on the development of the SLPPDS has informed a caregiver support element of a major programme addressing severely malnourished infants and children in Pujehun District (19).

### Mental health policy

Contextual understanding is critical for addressing the mental health and psychosocial support needs of Sierra Leone’s population (6). This means taking into account the country’s unique cultural, social, and economic context when developing policies and practices for mental health care. Community-based interventions and involving local communities in the development and implementation of mental health programmes are crucial for recognizing the significance of policies through the lens of societal norms. Effective community engagement enables co-creation of policy agendas, catering to addressing societal norms that could potentially create unintended consequences during the implementation stage. Such an approach highlights the importance of recognizing and respecting the cultural and social norms within a community while developing and implementing mental health programs. These considerations are vital for improving the overall effectiveness and sustainability of mental health interventions, promoting community involvement and ownership, and ultimately, reducing the burden of mental illness.

There are three major policy and practice implications of the contextual understanding for mental health care in Sierra Leone gained through the work described (and allied research). First, policies should prioritize community-based interventions, such as providing training for community health workers to identify and support individuals with mental health needs informed by the outputs from this study. Second, policies should prioritize the integration of mental health care into primary health care services to increase access to care. Third, policies should focus on reducing the stigma associated with mental illness, which can prevent individuals from seeking care. These three foci have shaped advocacy with the MoHS and, in collaboration with the MHCSL, with wider stakeholders, including at the national stakeholder briefing event convened in Freetown in April 2023 and with Special Adviser to the President of Sierra Leone on Mental Health.

## Data Availability

The raw data supporting the conclusions of this article will be made available by the authors, without undue reservation.

## Footnotes

1
https://ruhf.info/tools-resources/sierra-leone-psychological-distress-scale/
